# Quantitative EEG and dysautonomia in patients with temporal lobe epilepsy

**DOI:** 10.1186/s42494-025-00235-1

**Published:** 2026-01-05

**Authors:** Reem M. Gabr, Saly H. Elkholy, Amira A. Labib, Reham M. Shamloul, Micheal Baghdadi, Noha A. ElSawy

**Affiliations:** 1 Department of Neurology and Clinical Neurophysiology, Faculty of Medicine, Cairo University, Cairo, 11562 Egypt; 2 Radiology, Egyptian Ministry of Health, Cairo, 11516 Egypt

**Keywords:** Dysautonomia, Heart rate variability, Sympathetic skin response, Theta band power

## Abstract

**Background:**

The temporal lobe is considered as one of the important higher autonomic centers. It has been suggested that autonomic dysfunction could have a potential role in the pathophysiology of sudden unexpected death of epileptic patients (SUDEP). This study aimed to detect autonomic dysfunction in patients with temporal lobe epilepsy (TLE) using different electrophysiological tests and to correlate them with the SUDEP risk.

**Methods:**

This observational case–control study included 27 TLE patients and 27 age- and gender-matched controls. Detailed history and full clinical examination were performed. Brain MRI were done. Electrophysiological studies in the form of sympathetic skin responses (SSR), heart rate variability (HRV), as well as quantitative electroencephalography (QEEG) were performed. SUDEP risk was assessed using the SUDEP-7 inventory scale.

**Results:**

The mean age of the recruited patients was 28.3 ± 8.26 years, with mean seizure duration of 16.59 ± 7.82 years. Epileptogenic lesions were detected in 92.6% of patients, the most common of which was the mesial sclerosis (64%). About 18.6% of the patients achieved seizure control. The patients showed a significantly reduced root mean square of successive differences (RMSSD) at deep breathing (*P* = 0.003), significantly higher upper limb SSR amplitudes (*P* = 0.01), and a significantly higher theta band absolute power (TBP) (*P* = 0.046/0.036). Absolute fronto-central TBP significantly correlated with the SUDEP-7 scores (*r* = 0.484 and 0.421;* P* = 0.011/0.029). ROC curve analysis for QEEG showed sensitivity of 75% and 83.3% for Fz and Cz TBP respectively.

**Conclusions:**

Patients with TLE exhibit dysautonomia. Higher TBP as detected by QEEG reflects dysregulation of the higher autonomic centers, which may increase the risk of SUDEP. Thus, QEEG could serve as a sensitive, readily available biomarker for SUDEP risk screening.

**Supplementary Information:**

The online version contains supplementary material available at 10.1186/s42494-025-00235-1.

## Background

Temporal lobe epilepsy (TLE) is the most common form of focal epilepsy, representing 60% of all epilepsies [1]. Most autonomic seizures arise from the temporal lobe, highlighting the important role of the limbic system (including the insular cortex) among the higher cortical autonomic centers [2, 3]. In addition to the insular cortex, other structures are involved in the constellation of the autonomic network, such as the anterior cingulate cortex, amygdala, preoptic area, periaqueductal area, parabrachial nucleus, solitary tract nucleus, ventrolateral medulla, and medullary raphe nucleus [2, 4].

Autonomic manifestations could occur, either ictally or post-ictally, revealing the intimate connection between epileptic and autonomic networks [5]. Interestingly, autonomic dysfunction is thought to have a potential role in the pathophysiology of sudden unexpected death of epileptic patients (SUDEP), which is the most common cause of epilepsy-related deaths. It is reported to be highly associated with mesial temporal lobe epilepsy (mTLE) in particular [5–7]. This was evidenced by structural and functional imaging studies that demonstrated changes in the higher autonomic centers among patients with high SUDEP risk [5, 8].

The anterior cingulate cortex (ACC), as a part of the central autonomic network, is thought to have a crucial effect on cardiovascular functions, the most commonly reported autonomic manifestation among patients with epilepsy (PWE) [9]. Furthermore, electrical stimulation of the ACC is associated with heart rate and blood pressure changes [10]. Such changes were associated with the appearance of 3–8 Hz theta activity over the frontocentral region, as detected by EEG [11]. This “frontal midline theta activity” was studied using quantitative EEG techniques in a study conducted by Kubota and colleagues in 2001, which confirmed the presence of an interactive relationship between peripheral autonomic activities and the frontal cortical network [11].

This study aimed to detect dysautonomia among patients with TLE using different electrophysiological methods and to correlate this dysfunction with the SUDEP risk occurrence, as assessed by the SUDEP-7 inventory score.

## Materials and methods

### Study design

This observational case–control study received approval from the research ethics committee (REC) of the institute (MD-320 2020) before the start of the study and was also registered as a clinical trial (NCT06269822). Written informed consent was obtained from each patient. Patients’ personal data were hidden.

### Subjects and clinical assessment

Twenty-seven TLE patients were recruited from the epilepsy outpatient clinic at Cairo University Hospital from May 2022 to June 2023. The sample size was calculated depending on the primary outcomes (detecting autonomic dysfunction using electrophysiological tests in patients with TLE). According to a previous study by Atalar et al. [12] who used a heart rate variability test to detect dysautonomia, the mean difference in RMSSD (during deep inspiration) between patient and control groups was 65.27, with respective standard deviations of 100.87 and 77.95. To achieve a 90% confidence level, margin of error of 10% (type I error = 0.1) and 80% power (type II error = 0.20); a minimum sample size of 24 participants was required in each group. The sample size was calculated using MedCalc software version 12.4.

The patients received a diagnosis of TLE depending on the clinical semiology and the inter-ictal temporal epileptiform discharges. Seizure types were further classified according to the latest ILAE classification [13]. Brain MRI was performed for each patient to identify the presence of epileptogenic lesions. Subjects aged > 18 years of either gender were included in the study. Twenty-seven age- and gender-matched healthy subjects were included as the control group.

We excluded patients with any identifiable disease that may compromise the autonomic nervous system (ANS) function (such as diabetes mellitus, multiple sclerosis, and Parkinson's disease) and patients treated with drugs known to affect the ANS (including phenytoin, anti-arrhythmic drugs, and oral contraceptives). Patients who had seizures 8 h before or 1 h after the electrophysiological tests were also excluded from the study.

A detailed history, including seizure diary documentation, was obtained from each patient. Autonomic manifestations were assessed using a standardized autonomic questionnaire based on a study addressing autonomic dysfunction in PWE [12] (Appendix I in Supplementary Information). The SUDEP risk was assessed with the “SUDEP-7 inventory score,” a subsequent score was given for each patient. This validated weighted score assesses SUDEP risk based on seven risk factors, such as seizure frequency [6, 15]. Routine laboratory tests were performed, including metabolic screening (such as HbA1c, renal and hepatic function tests) to confirm compliance with the exclusion criteria.

A general clinical examination was performed, including measuring blood pressure during the supine position and three minutes after erect position, as a bedside autonomic test to detect the presence of orthostatic hypotension.

### Electrophysiological studies assessing the peripheral domain of the ANS

The electrophysiological tests were conducted in the afternoon (2–6 pm) for all recruited subjects to avoid the effect of circadian rhythm on the ANS. Patients were instructed not to do heavy exercise and to avoid ingesting heavy meals and caffeine at least 2 h before the tests.

#### Heart rate variability (HRV)

The HRV test was conducted using a Natus Viking EMG machine 672–003800 REV.08, Wisconsin, USA. The active electrode was placed on the 5th intercostal space (apex of the heart), the reference electrode was placed on the clavicle, and the ground electrode was around the wrist.

After a 10-min rest period in the supine position, the recording of QRS complexes was initiated for one minute, during which 12 recordings were analyzed. Then, another one-min recording of regular breathing at a rate of 6-breaths/min (with patients following the metronome displayed on the screen) was analyzed. The trigger line was manually adjusted to trigger the R-wave of QRS complexes. The traces were reviewed before analysis to exclude any artifactual traces.

The root mean square of successive differences, the most important time-domain parameter in the ultra-short recordings (< 5 min), was automatically calculated in milliseconds (ms). This time domain parameter is relatively unaffected by respiration and thus serves as an index of the vagal activity [16].

#### Sympathetic skin response (SSR)

The SSR was recorded using Nihon-Khoden EMG Neuropack M1 MEB−9200 machine (Tokyo, Japan). Responses were simultaneously elicited from the left hand and foot while stimulating the right median nerve at the wrist. The active electrodes were placed on the palm of the left hand and the sole of the foot, while the reference electrodes were placed on the dorsal surfaces of the left hand and foot. The ground electrode was placed on the left wrist.

An electrical stimulus of 0.2 ms duration and an intensity range of 20–30 mA was delivered randomly, at a minimum inter-stimulus interval of > 30 s. Two responses were elicited to confirm reproducibility; the response with the larger amplitude was selected.

Then, the response latency, measured from stimulus artifact onset to the first deflection of the potential from baseline in ms, and peak to peak amplitude in μv, were recorded [17].

### Quantitative EEG (QEEG) to assess the central domain of the ANS

EEG was performed using EBNeuro Galileo NT EEG machine (Firenze, Italy) while the subject lay comfortably in the dorsal recumbent position in a semi-illuminated quiet room with eyes closed. The electrodes were applied according to the international 10–20 system, with 2 additional extracerebral electrodes placed on the anterior chest for ECG recording.

Data were recorded using a sampling rate of 200 or more with filter setting ranges 1–70 Hz. An artifact-free epoch of 10-s duration was selected, and the theta (4–8 Hz) absolute power at the Fz and Cz locations was quantitatively assessed using the Fast Fourier transform (FFT) [11, 18].

### Magnetic resonance imaging (MRI)

The MRI protocol for the assessment of epilepsy patients includes:

1. T1-weighted volumetric dataset acquired in oblique coronal orientation orthogonal to the axis of the hippocampus (slice thickness 0.9–1 mm).
2. Oblique coronal spin echo sequences and heavily T2-weighted sequences perpendicular to the hippocampus.
3. Fluid attenuation inversion recovery (FLAIR) sequences in coronal and axial axes [19].

### Statistical methods

The data were coded and entered using the statistical package for the Social Sciences (SPSS) version 28 (IBM Corp., Armonk, NY, USA). Quantitative data were summarized using mean, standard deviation, median, minimum, and maximum, and categorical data were described as frequency (count) and relative frequency (percentage).

Quantitative variables were compared using the non-parametric Kruskal–Wallis and Mann–Whitney tests [20]. A Chi-square (χ2) test was performed to compare categorical data. Fisher’s test was used when the expected frequencies were less than 5 [21]. Quantitative variables were compared using the Spearman’s correlation coefficient [22]. A *P*-value of less than 0.05 was considered statistically significant. The diagnostic performance of QEEG and HRV tests, for SUDEP risk detection, was assessed using receiver operating characteristic (ROC) curves.

## Results

### Demographic data

The mean age of the recruited patients was 28.3 ± 8.26 years, and that of controls was 32.07 ± 9.58 (*P* = 0.163). Females accounted for 56% of the patients and 52% of the controls (*P* = 0.785).

### Clinical data

The mean duration of epilepsy was 16.59 ± 7.82 years. Structural etiologies accounted for 92.6% of the patients. About 18.6% (5/27) of the patients were controlled, being seizure-free for over a year. Further clinical characteristics of the patients are demonstrated in Table 1. Autonomic manifestations were reported in 51.9% (14/27), with cardiovascular being most common (57%; 8/14), followed by sudomotor, gastrointestinal, and genitourinary symptoms. Most patients (78.57%; 11/14) reported > 1 symptom.

**Table 1:** Clinical Characteristics of the patients

	No. of patients
Seizure classification	
Focal unaware	27/27 (100%)
Focal unaware to BTC	8/27 (29.6%)
Auras	12/27 (44%)
Automatisms	14/27(51.9%)
MRI Findings	
Mesial sclerosis	16/25 (64%)
Other lesions (cortical dysplasia, old ischemic insult, subependymal heterotropia cortical thinning and cavernomas)	9/25 (36%)
Seizure etiology	
Structural	25/27 (92.6%)
Unknown	2/27 (7.4%)
Patient management	
Polytherpay	25/27(92.6%)
Pharmaco-resistant	22/27(74.1%)

*BTC* bilateral tonic clonic seizures, *ASMs* Anti-seizure medications

**Fig. 1:**
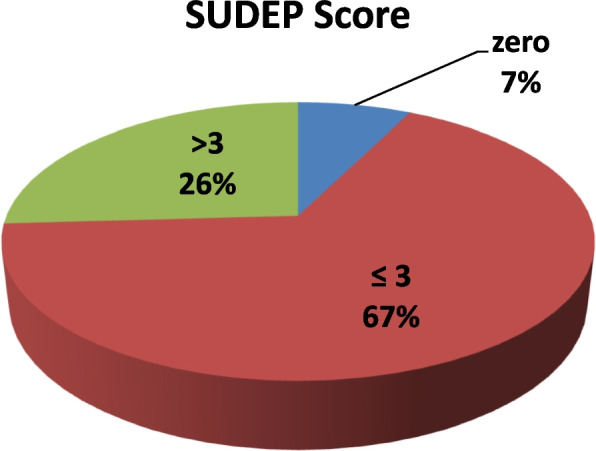
SUDEP score among the allocated patients. *SUDEP Sudden unexpected death of epileptic patients*

Regarding the SUDEP-7 inventory score, 67% of the patients (18/27) had scores of ≤ 3. Meanwhile, 7 patients (26%) had SUDEP scores > 3, and the remaining two patients (7%) had zero SUDEP scores and were on monotherapy with pharmaco-responsiveness to their medication (Fig. 1).

### Dysautonomia as detected by electrophysiological tests

**Fig. 2:**
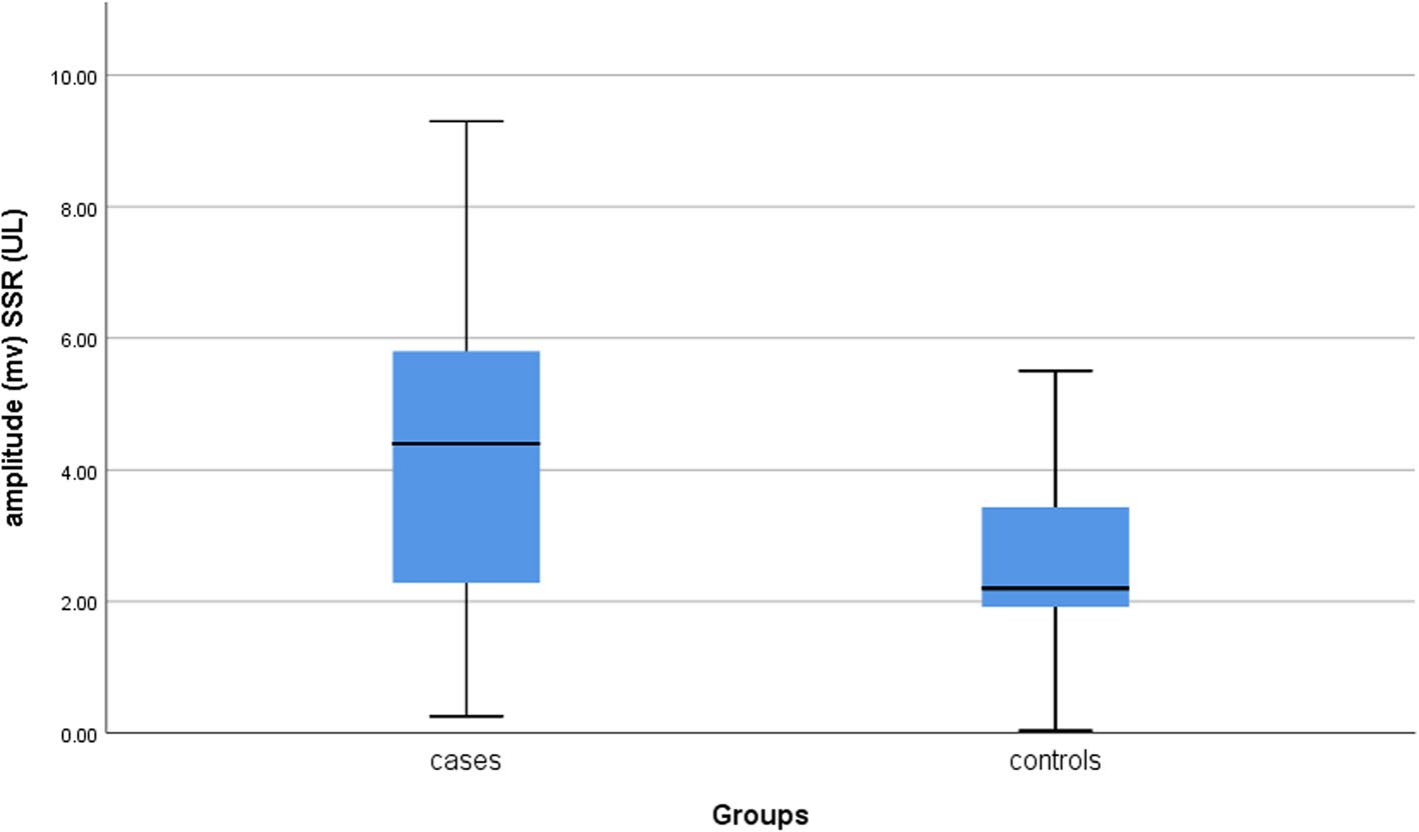
Significantly higher upper limb SSR amplitude responses of the patients compared to the controls (*P = *0.001*)

A higher sympathetic tone was expressed among the patients than the matched controls by the significantly higher amplitudes of upper limb SSR responses (*P* = 0.01). Moreover, relatively delayed latencies in both the upper and lower limbs and higher amplitudes in the lower limbs were detected, yet these parameters did not show statistically significant differences (Fig. 2).

**Fig. 3:**
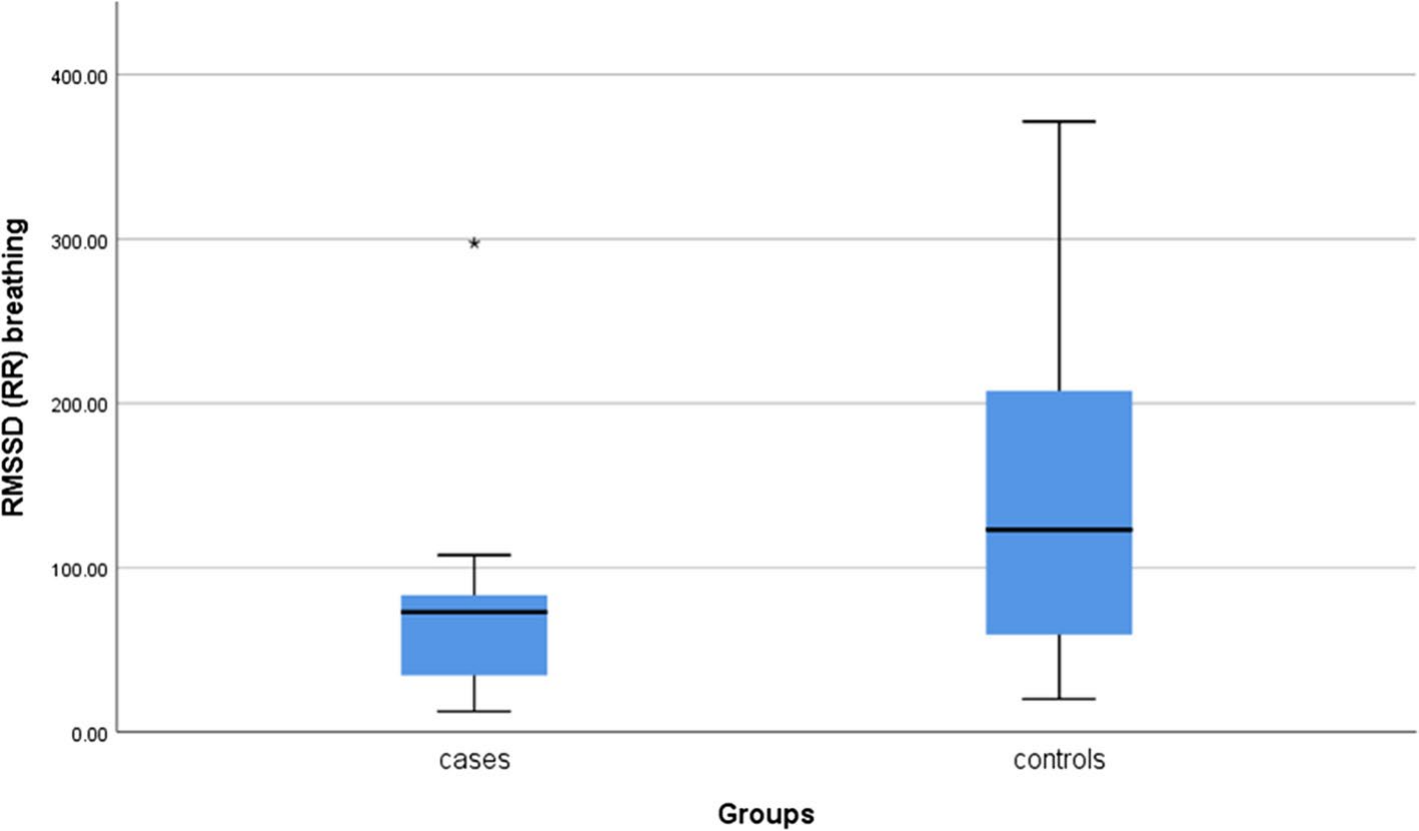
Significantly reduced RMSSD during deep breathing (*P = 0.003). *RMSSD Root mean square of successive differences; RR R-R interval test *

HRV analysis, as an index of parasympathetic function, revealed significantly reduced RMSSD among the patients (*P* = 0.003; Fig. 3).

The QEEG studies showed a significantly higher absolute theta band power at both Fz and Cz in the TLE patients (*P* = 0.046 & 0.036; Table 2).

**Table 2:** QEEG absolute power at Fz and Cz among the cases and controls

	**Patients (*n* = 27)**					**Controls (*n* = 27)**					***P value***
	**Mean**	**SD**	**Median**	**Min**	**Max**	**Mean**	**SD**	**Median**	**Min**	**Max**	
Fz (Absolute power)	23.51	17.90	18.65	4.89	62.91	15.90	13.49	11.29	2.99	52.66	0.046*
Cz (Absolute power)	20.01	19.08	14.64	2.26	70.49	10.48	9.34	7.77	1.80	45.13	0.036*

^*^ Significant *P-values*

*Mann Whitney test*

### Diagnostic performance of QEEG and HRV tests in detecting SUDEP risk

**Fig. 4:**
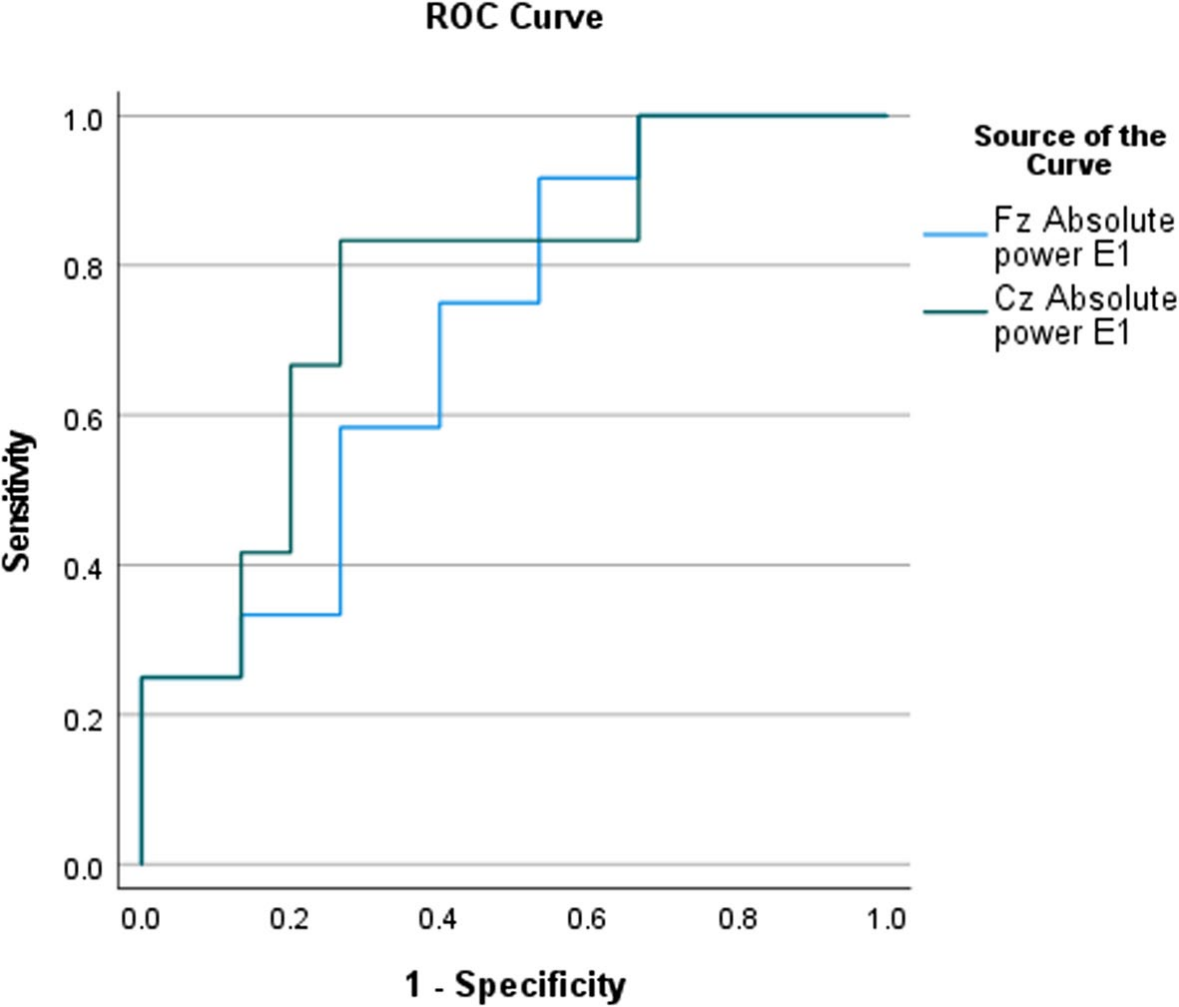
ROC analysis of QEEG

**Fig. 5:**
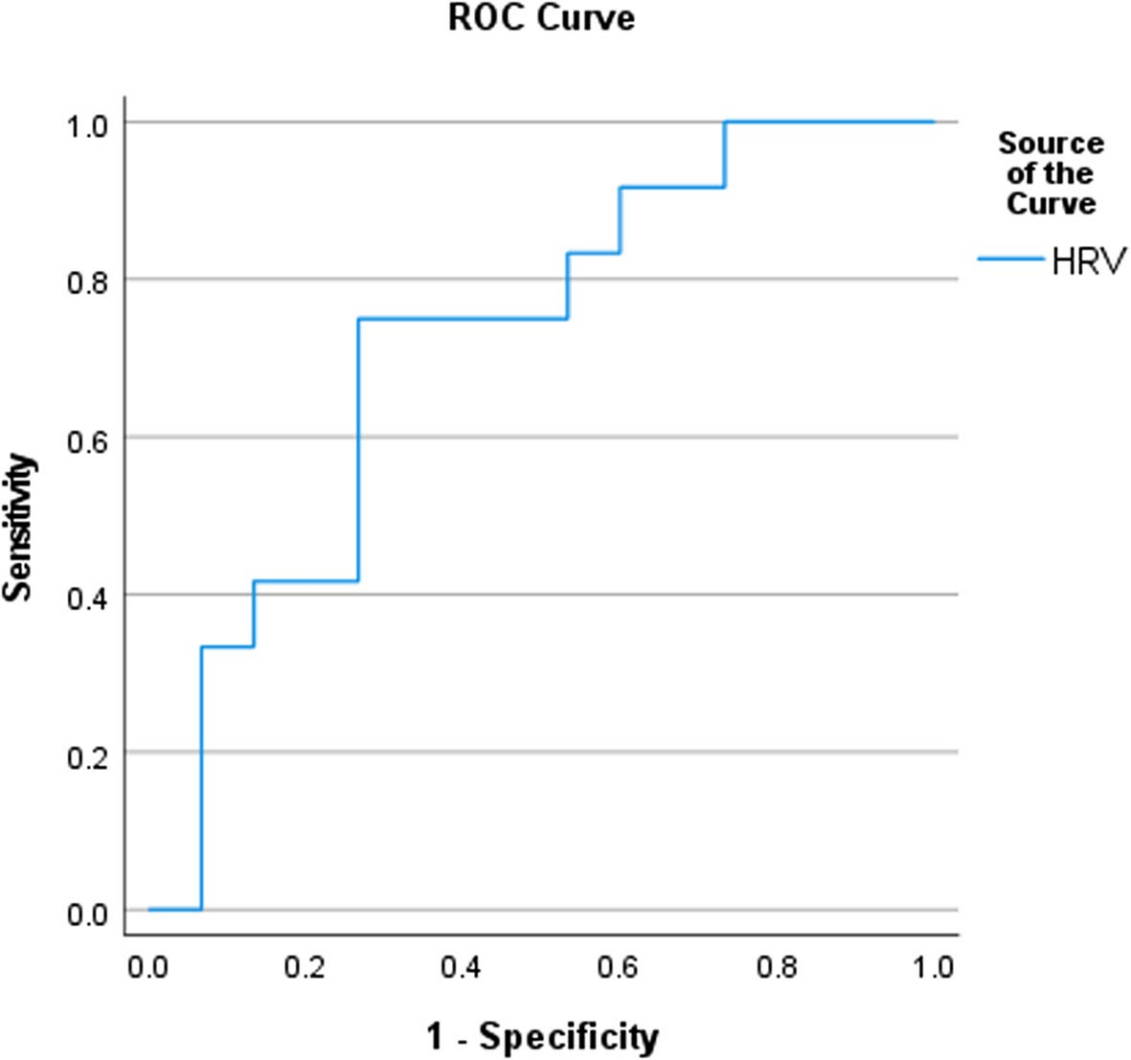
ROC analysis of HRV test. *HRV Heart Rate Variability *

ROC curve analysis for QEEG showed cut-off values of 16.89 and 14.665 for the Fz and Cz electrodes with sensitivity of 75% and 83.3%, respectively (Fig. 4). While the ROC analysis for HRV test revealed a cut-off value of < 71.845, with a sensitivity of 75% (Fig. 5).

### Correlation between QEEG theta band power and ASMs

No significant correlation was found between number of anti-seizure medications and fronto-central theta power (Table 3).

**Table 3:** Correlation between number of ASMs and theta band power

Electrode	Parameter	**Number of ASMs**	***P-value***
Fz (Absolute power)	**Correlation coefficient**	0.125	0.533
Cz (Absolute power)	**Correlation coefficient**	0.234	0.241

*ASMs Anti-seizure medications*

*Spearman correlation*

### Correlation between the SUDEP score and electrophysiological tests

Interestingly, a significant positive correlation was found between the increased theta band absolute power at Fz and Cz locations and the SUDEP score (Table 4).

**Table 4:** Correlation of absolute theta band power and SUDEP score

SUDEP score		
	**Correlation coefficient**	***P value***
**Fz (Absolute power)**	0.484	0.011*
**Cz (Absolute power)**	0.421	0.029*

*SUDEP* *Sudden unexpected death of epileptic patients*

^*^ Significant *P-values*

*Spearman correlation*

No significant correlation was found between either SSR or RMSSD and the SUDEP score.

## Discussion

There is a growing interest in studying the functional integrity of the ANS among PWE [14, 23–27]. Despite variability in the study designs and modalities used, a consensus of decreased vagal tone and presumably increased sympathetic tone during the inter-ictal period among PWE was reached [16, 27].

A significantly higher SSR amplitude of the upper extremity was noted among patients. This non-length-dependent pattern, contrasting with peripheral neuropathy disorders, was confirmed in other studies [12, 23]. Such findings may add strength to the postulation of the higher sensitivity of the upper limbs’ SSR; thus, the suggestion of placing the seizure detector devices on the upper limbs is made [25, 26].

Though the principal markers suggesting SSR abnormality are still controversial, the latency measurements are of limited value, as they could be affected by abnormalities in either the efferent or the afferent of the SSR reflex. Moreover, latency is subject to circadian variation. On the other hand, other authors prefer latency measurements, assuming them to be less variable than amplitude [17, 28].

Though both parameters were assessed in this study, we assumed that the amplitude would be more valuable, supporting our inquiry in detecting sympathetic over-activity.

The RMSSD is the most reliable index for ultra-short recordings (< 5 min) [27, 29]. The significantly reduced RMSSD during deep breathing among PWE suggested a decreased cardiovagal activity [30]. Moreover, there was a trend towards reduced RMSSD at rest, yet it did not reach a statistical significance. This agrees with other studies that only observed decreased cardiovagal activity during deep breathing [15, 25].

A recent emerging theory postulated that epilepsy-related autonomic changes were rooted in the same alterations within the central activity; this may explain the relationship between dysautonomia and epilepsy [31]. Thus, the assignment of midline theta band power (TBP), using QEEG, in the allocated patients was proposed to provide valuable information supporting this hypothesis.

A significantly higher TBP at both Fz and Cz scalp electrodes was statistically confirmed in patients with epilepsy. Similarly, a mild to moderate increase in the TBP was reported among PWE in previous studies; moreover, some researchers considered it an epileptic trait [32–34].

This frontal midline theta activity is thought to originate from the ACC, which is confirmed by studies using magnetoencephalograph (MEG); it is specifically related to the sympathetic nervous system activity [35–37]. In another study, supporting its relation with the sympathetic function, such frontal midline TBP was positively correlated with the cardiac sympathetic index [18].

Interestingly, a recent study using functional MRI demonstrated higher autonomic centers dysregulation, showing functional connectivity alterations between different cortical structures, including the cingulate cortex and other structures [38].

Most patients (74.1%) were on polytherapy, and the majority (92.6%) were drug-resistant. As they were recruited from a tertiary hospital, addressing the effect of ASMs on the frontocentral theta band activity was crucial. No significant correlation was found between the number of ASMs and TBP.

This was established in the literature since late 1990s by Díaz and colleagues, who noted that there were no significant differences between the treated and the untreated PWE regarding QEEG alterations [32]. Later in 2015, *Abdel *et al. found significantly increased TBP in the midline and parasagittal regions among non-medicated patients compared to the medicated group, suggesting that these changes occur independent of the ASMs’ effect [39].

Furthermore, the literature data addressing the effect of ASMs on autonomic function, as assessed by electrophysiological tests, is still controversial. Some studies assume that carbamazepine may affect cardiac autonomic function, whereas other researchers argue against the presence of any correlation between ASMs and autonomic function as assessed by HRV and electrodermal activity (EDA), depending on comparative studies of treated and untreated PWE [40]. This was further confirmed by a recent meta-analysis finding no differences between ASM-treated ASM-free patients [41].

To our knowledge, the correlation between the interictal sympathetic findings, as expressed by the SSR abnormalities and the SUDEP score, has not been discussed previously. This study found no significant correlation between the SUDEP score and SSR parameters. Additionally, no correlation was found between the HRV and the SUDEP risk. This comes in agreement with other studies, including a recent systematic review [16, 42–44].

Meanwhile, reduced RMSSD among PWE with higher SUDEP risk was observed in other studies [22, 45]; however, such studies included both focal and generalized types of epilepsy and applied different HRV protocols with longer recording durations (> 5 min). The inconsistent results regarding the correlation between HRV and SUDEP risk could be explained by differences in the methodological approaches across studies, particularly the lack of a standardized HRV protocol.

The lack of correlation between SSR, a peripheral autonomic nervous system test, and QEEG, which reflects higher autonomic center function, could be attributed to the fact that dysautonomia in PWE is caused by alterations of central autonomic network rather than peripheral dysautonomia. Other possible explanations could be the low sensitivity of SSR test in detecting dysautonomia.

Interestingly, a statistically significant positive correlation was found between increased TBP and the SUDEP score. The risk of SUDEP in PWE could be assessed by four different scores: SUDEP-7 inventory, SUDEP-3 inventory, SUDEP-CARE score and Kempenhaeghe scores (only the first three are validated) [46]. We used the SUDEP-7 inventory score as it is the most commonly used metric in the literature and encompasses most SUDEP risk factors. Moreover, the ROC curve analysis of QEEG revealed comparable sensitivity to the HRV test, a previously recognized potential biomarker of SUDEP risk.

Such findings pose a suggestion that QEEG could be used as a promising, non-invasive, readily available, and inexpensive biomarker for SUDEP risk assessment. Interestingly, this finding was supported by a recent study that included patients with Dravet syndrome [47]. Our study is considered one of the earliest studies addressing the TBP in association with SUDEP risk.

This study has some limitations. First, the SUDEP risk was not assessed by an actual event, though the SUDEP-7 inventory score is a valuable weighted score derived from a large cohort study. Also, other QEEG techniques, such as coherence, together with functional MRI studies, are recommended to be applied to ensure the disruptions within the central autonomic networks. Although the sample size was calculated depending on the primary outcome, larger multi-center cohort studies are recommended to confirm the other findings. Long-term follow-up studies are also recommended to address the long-term changes in the autonomic function.

## Conclusions

Patients with TLE exhibit inter-ictal peripheral autonomic dysfunction characterized by decreased vagal tone and increased sympathetic activity. Furthermore, dysregulation of the higher autonomic centers was implicated in terms of the QEEG parameters, which could potentially serve as a sensitive biomarker for SUDEP screening. This is one of the earliest studies addressing the association between the fronto-central theta band power and SUDEP risk.

## Supplementary Information

Supplementary Material 1.

## Data Availability

The datasets generated for this study are available upon request from the corresponding author.

## Abbreviations

ACC: Anterior cingulate cortex
ANS: Autonomic nervous system
ASMs: Anti-seizure medications
EDA: Electrodermal activity
FFT: Fast-fourier transform
FLAIR: Fluid attenuation inversion recovery
HRV: Heart rate variability
MEG: Magentoencephalography
MRI: Magnetic resonance imaging
mTLE: Mesial temporal lobe epilepsy
PWE: Patients with epilepsy
QEEG: Quantitative electroencephalography
RMSSD: Root mean square of successive differences
ROC: Receiver operating characteristic
SPSS: Statistical package for the Social Sciences
SUDEP: Sudden unexpected death of epileptic patients
TBP: Theta band power
TLE: Temporal lobe epilepsy
