# Supplementary material for: Quantitative EEG and dysautonomia in patients with temporal lobe epilepsy
Source: Acta Epileptol. 2026 Jan 5;8:2. doi: 10.1186/s42494-025-00235-1 (PMC12766958; doi:10.1186/s42494-025-00235-1)
Supplement: Supplementary file 1 — Supplementary Material 1. [file 42494_2025_235_MOESM1_ESM.docx]

**Appendix I (Autonomic Questionnaire):**

| **Cardiovascular** | Palpitations  Orthostatic intolerance  Dizziness |
| --- | --- |
| **Gastro-intestinal** | Post-prandial Fullness / bloating  Diarrhea / Constipation  Fecal incontinence |
| **Sudomotor** | Hyperhidrosis/Hypohidrosis  Skin color changes; pallor or erthyma |
| **Genitourinary** | Incontinence  Nocturia  Erectile dysfunction/ loss of lipido |

Each patient was asked for the presence/absence of each manifestation listed above and then the data of each patient was collected as presence/absence of autonomic manifestations

**ROC curve details for QEEG**

| **Test result variable(s)** | **AUC** | ***P* value** | **95% CI** | |  |  |  |
| --- | --- | --- | --- | --- | --- | --- | --- |
|  |  |  | **Lower bound** | **Upper bound** | **Cutoff value** | **Sensitivity %** | **Specificity %** |
| **Fz absolute power** | 0.711 | 0.034 | 0.516 | 0.906 | 16.895 | 75 | 60 |
| **Cz absolute power** | 0.772 | 0.003 | 0.591 | 0.954 | 14.665 | 83.3 | 73.3 |

AUC Area under curve; CI Confidence interval

**ROC curve details for HRV test**

| **Test result variable(s)** | **AUC** | ***P* value** | **95% CI** | |  |  |  |
| --- | --- | --- | --- | --- | --- | --- | --- |
|  |  |  | **Lower bound** | **Upper bound** | **Cutoff value** | **Sensitivity %** | **Specificity %** |
| **HRV** | 0.722 | 0.026 | 0.526 | 0.918 | < 71.845 | 75 | 73.3 |

AUC Area under curve; CI Confidence interval; HRV Heart rate variability
